# In vivo imaging of the tonoplast intrinsic protein family in Arabidopsis roots

**DOI:** 10.1186/1471-2229-9-133

**Published:** 2009-11-18

**Authors:** Stefano Gattolin, Mathias Sorieul, Paul R Hunter, Roman H Khonsari, Lorenzo Frigerio

**Affiliations:** 1 Department of Biological Sciences, University of Warwick, Coventry CV4 7AL, UK

## Abstract

**Background:**

Tonoplast intrinsic proteins (TIPs) are widely used as markers for vacuolar compartments in higher plants. Ten TIP isoforms are encoded by the Arabidopsis genome. For several isoforms, the tissue and cell specific pattern of expression are not known.

**Results:**

We generated fluorescent protein fusions to the genomic sequences of all members of the Arabidopsis TIP family whose expression is predicted to occur in root tissues (TIP1;1 and 1;2; TIP2;1, 2;2 and 2;3; TIP4;1) and expressed these fusions, both individually and in selected pairwise combinations, in transgenic Arabidopsis. Analysis by confocal microscopy revealed that TIP distribution varied between different cell layers within the root axis, with extensive co-expression of some TIPs and more restricted expression patterns for other isoforms. TIP isoforms whose expression overlapped appeared to localise to the tonoplast of the central vacuole, vacuolar bulbs and smaller, uncharacterised structures.

**Conclusion:**

We have produced a comprehensive atlas of TIP expression in Arabidopsis roots, which reveals novel expression patterns for not previously studied TIPs.

## Background

Tonoplast intrinsic proteins (TIPs) are a subfamily of aquaporins, small integral membrane proteins belonging to the major intrinsic protein (MIPs) family. Aquaporins form channels that facilitate the movement of water, small uncharged solutes (glycerol, urea, boric acid, silicic acid, hydrogen peroxide) and gases (ammonia, carbon dioxide) across biological membranes. (For recent reviews see [1,2]). TIPs have been either detected, or predicted to localise, to the tonoplast [3].

The Arabidopsis genome encodes 10 TIP isoforms [4], further classified into five subgroups: three γ-TIP (TIP1), three δ-TIP (TIP2), the seed-specific α- and β-TIP (TIP3;1 and TIP3;2), one ε-TIP (TIP4;1) and one ζ-TIP (TIP5;1).

Several TIP isoforms have been studied in detail as regards their expression [3,5,6] and function [7,8]. TIPs have also been widely employed as intracellular markers for vacuolar biogenesis and identity. Immunofluorescence experiments in root tips and mature embryos of different plant species led to the identification of separate vacuolar compartments within the same cell [9-13]. These experiments indicated an association of γ-TIP (TIP1;1) with vegetative, lytic-type vacuoles and of α-TIP (TIP3;1) and δ-TIP (TIP2;1) with protein storage vacuoles. The detection of different TIP isoforms on separate tonoplasts provided evidence for multiple, functionally different vacuolar compartments within plant cells (reviewed in Frigerio et al, 2008). Recently we compared expression of TIP3;1 and TIP1;1 in Arabidopsis and found minimal overlap in the timing of their expression, with TIP3;1 being abundant in embryos of mature seeds and sharply declining during seed germination, to be replaced by TIP1;1 [14]. The latter was not present in root tips, thus raising some doubt as to the applicability of these particular isoforms as vacuolar markers in Arabidopsis [5,14]. As the investigation was limited to the three TIP isoforms against which peptide antibodies were raised for the immunofluorescence studies [10], the possibility remained that other TIP family members with similar immunoreactivity may be present in different vacuoles within Arabidopsis root tissues. Indeed, the tissue-specificity of expression of some TIP family members has not yet been investigated in detail.

In this report we have mapped the expression of every Arabidopsis TIP isoform that is predicted to be present in root tissues by transcriptomic analysis [15]. This excludes TIP3;1 and TIP3;2 (α and β-TIP), which have seed-specific expression patterns [14,16]; Gattolin and Frigerio, unpublished), and both TIP1;3 (γ-TIP3) and TIP5;1 (ζ-TIP), which are predicted by bioinformatic analysis to be expressed solely in floral organs and pollen [15-17].

Our results indicate that expression of some TIP isoforms under their native promoters is remarkably tissue and cell-specific. In general, when multiple isoforms are co-expressed in the same cell, they appear to localise mainly to the tonoplast of the central vacuole. Our identification of the sites of expression of every TIP isoform paves the way to understanding TIP specialisation and function in Arabidopsis root tissues.

## Results

**Figure 1:**
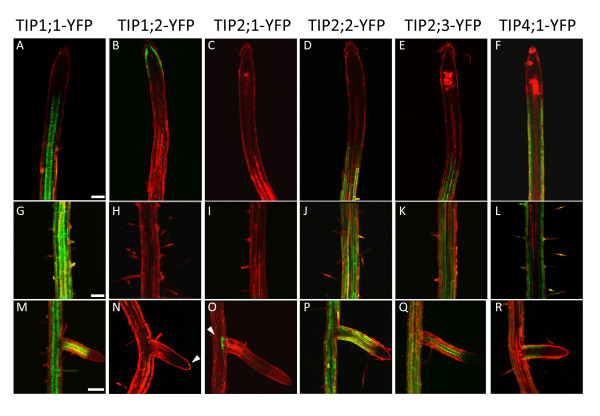
**Expression patterns of TIP isoforms in Arabidopsis roots**. 8-day old roots from the indicated transgenic lines were excised, stained with propidium iodide for 2 min and visualised by CLSM. The images show representative results for each construct. The signals from YFP (green) and propidium iodide fluorescence (red) are merged. Top panels: single optical sections of the root tips, Middle panels: single optical sections of root differentiation zones. Bottom panels: maximal projection of 16 optical z sections (4 μm step-size) through mature root axes and young lateral roots. Scale bars: 100 μm.

In addition to the fluorescent TIP reporters we generated previously for TIP1;1 (γ-TIP1; At2g36830) and TIP2;1 (δ-TIP1; At3g16240) [14], we cloned the genomic sequences of not previously studied isoforms: TIP1:2 (γ-TIP2; At3g26520), TIP2;2, TIP2;3 (δ-TIP2 and δ-TIP3; At4g17340 and At5g47450) and TIP4;1 (ε-TIP; At2g25810). We produced chimeric constructs in which either YFP or monomeric RFP were fused in frame to the C-terminus of each TIP genomic sequence (including their promoter regions, 5' UTR and introns), and generated transgenic plants which were analysed for TIP-XFP expression patterns by confocal laser scanning microscopy (CLSM). We first observed TIP-YFP expression at low magnification. 8-day old roots from seedlings expressing individual YFP-tagged TIPs were stained with propidium iodide and analysed by CLSM. With the possible exception of TIP1;2, no YFP-tagged TIP isoforms yielded a detectable signal in the root cap or meristem (Fig. 1, panels A to F). In general, TIP-YFP expression initiates at the elongation zone. While TIP1;1-YFP and TIP4;1-YFP are detectable from the base of the elongation zone (panels A and F), TIP2;2-YFP and TIP2;3-YFP expression is first observed at the zone of transition with the differentiation zone (panels D and E). The onset of fluorescence occurs in different cell types depending on the isoforms. TIP1;1-YFP is initially visible in endodermal cells, before extending to every cell type in the differentiation zone (Fig. 1, compare panels A and G). This pattern is faithfully replicated in lateral roots (Fig. 1M). TIP1;1 expression is strongest at the differentiation zone (Fig. 1G). TIP2;2-YFP becomes first detectable in the cortex and epidermis, but its expression extends to the pericycle as the root matures (Fig. 1, compare panels D and J). TIP2;3-YFP has a similarly widespread distribution in more mature root axes but its expression initiates in the pericycle, then extends to cortex and epidermal cells (Fig. 1, panels E and K). Again, the initial expression patterns of TIP2;2 and TIP2;3 are mirrored in the lateral roots (Fig 1, panels P and Q). In contrast to the previous isoforms, TIP4;1-YFP is only expressed in epidermal and (less strongly) in cortical cells of the differentiation zone (Fig. 1, panels F and L), with fluorescence decreasing in more mature parts of the root where lateral roots emerge (Fig. 1R).

In the case of TIP1;2, expression seems to be exclusively limited to the root cap and the columella (Fig. 1B). A very limited YFP signal can also be detected in the same region of the young lateral root (Fig 1N, arrowhead) and in older lateral roots (Additional file 1A).

Perhaps the most remarkable expression pattern observed is that of TIP2;1, which in 8-day old roots is only detectable in a small region at the base of the lateral roots (Fig. 1O, arrowhead).

**Figure 2:**
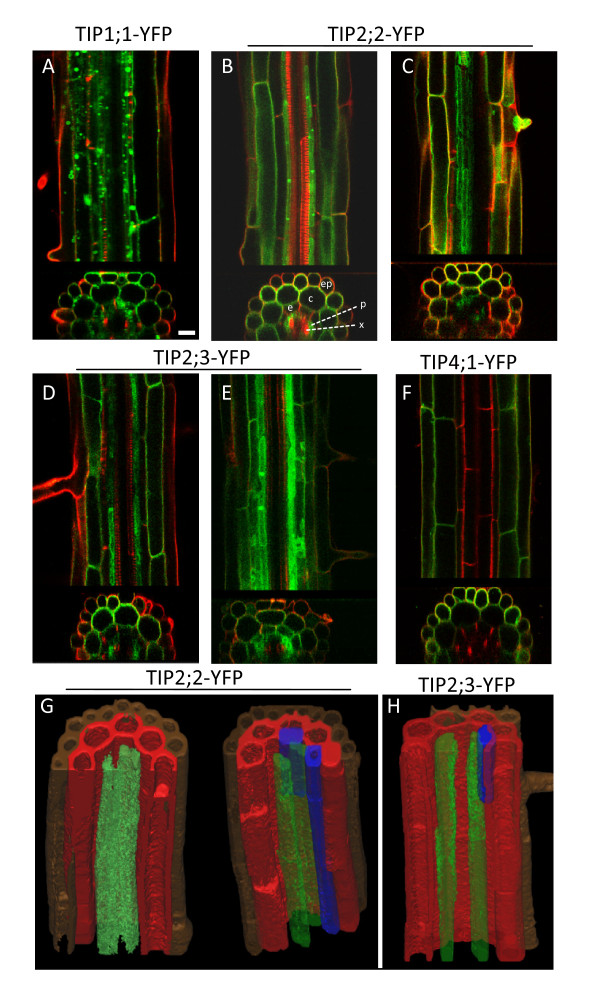
**Cell-type specificity of TIP-YFP expression in the root axis**. 8-day old roots from the indicated transgenic lines were excised, stained with propidium iodide for 2 min and visualised by CLSM. Stacks of 80 optical z sections (1 μm step-size) were collected from root axes at the differentiation zone. The images show representative results for each construct. A to F: for each panel, the top section shows a single xy optical section, and the bottom section shows the xz projection of the whole image stack, revealing the cross section of the root axis. The signals from YFP fluorescence (green) and propidium iodide fluorescence (red) are merged. G and H: the YFP fluorescence trace from representative image stacks for the indicated transgenic lines was reconstructed, segmented and rendered in 3D with Mimics 12.1. The different tissues are colour-coded as follows: brown, epidermis; red, cortex; blue, endodermis; green, pericycle. Ep, epidermis; c, cortex; e, endodermis; p, pericycle; x, xylem. Scale bar: 20 μm.

Having identified the general patterns of expression of the different isoforms at low magnification, we then studied the cell-specificity of TIP-YFP expression in more detail. We analysed propidium iodide -stained roots by CLSM by performing optical z-sections through differentiation zones at 63× magnification (Fig. 2).

TIP1;1-YFP is clearly expressed in epidermis and cortex, but its expression is particularly strong in the endodermis and pericycle (Fig. 2A). Here TIP1;1-YFP highlights numerous bright circular structures in the lumen of the central vacuole. We hypothesise these are vacuolar 'bulbs', which have previously been described as tonoplast invaginations, which occur independently of the ectopic expression of XFP-tagged membrane proteins, and where TIP1;1-GFP is concentrated [18,19]. It is however difficult in some cases to observe a continuity between these structures and the central vacuole tonoplast.

At higher magnification, the overlapping patterns of expression of TIP2;2-YFP and TIP2;3-YFP are confirmed. Both are present in pericycle cells, particularly in the rows of pericycle cells that form the xylem poles [20]. Both TIP-YFPs tend to be absent from the endodermis (Fig. 2, panels B and D), although we could detect discontinuous endodermal expression at various positions along most root axes (Fig. 2, panels C and E; and highlighted in blue in panels G and H).

In contrast to the previous isoforms labelling inner root cell layers, TIP4;1 expression is clearly restricted to the root epidermis and cortex, with no signal detectable in the inner layers (Fig. 2F).

### Localisation of TIP1;1 and TIP1;2

**Figure 3:**
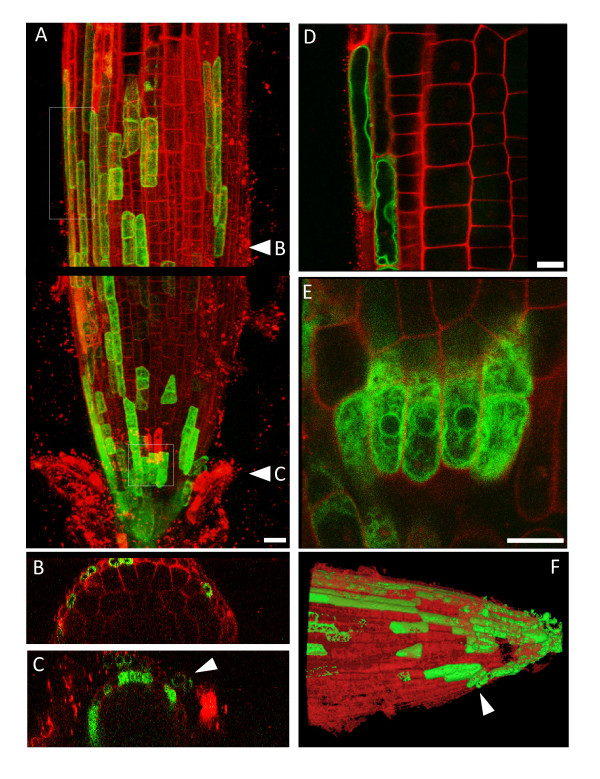
**YFP-TIP1;2 is expressed in the root cap**. 8-day old roots from the indicated transgenic lines were excised, stained with propidium iodide for 2 min and visualised by CLSM. Stacks of 80 optical z sections (1 μm step-size) were collected from root tips. The images show a representative result for this construct. The signals from YFP fluorescence (green) and propidium iodide fluorescence (red) are merged. A: maximal 3D projection of the root tip at the base of the elongation zone. The image shows two adjacent z-stacks of the same root, separated by a black line. B and C: xz projections of the image stack in panel a, revealing two cross-sections of the root axis, taken in the regions of the root indicated by the arrowheads in A. D and E: the regions indicated by dotted boxes in A were observed at high magnification. Single optical sections are shown. Note YFP-TIP1;2 in the ER of young root cap cells and in the tonoplast of root cap cells closer to the elongation zone. F: The fluorescent traces from YFP (green) and propidium iodide (red) from the image stack in panels A were reconstructed, segmented and rendered in 3D with Mimics 12.1. Scale bars: (a), 20 μm; (d) and (e), 10 μm.

Having analysed the TIPs with the broadest expression patterns, we focussed on the two TIPs which seem to have a more limited expression range in roots. TIP1;2-YFP presented a patchy distribution in cells of the root cap (Fig 1B). To ascertain that this was not an artefact due to expression of our chimeric gene, we also generated a construct (YFP-TIP1;2) where YFP was fused downstream of the promoter and 5'UTR and in frame with the 5' of the TIP coding sequence. In transgenic plants, YFP-TIP1;2 presents a similar expression pattern to TIP1;2-YFP, thus ruling out YFP fusion artefacts (compare Fig. 3A with Fig. 1B). Expression is confined to the columella and the lateral root cap [21], with the labelled cells disappearing at the boundary with the elongation zone (Fig. 3A). Some of the labelled cells are in the process of detaching from the root (Fig. 3, panels C and F, arrowheads), suggesting that they may correspond to 'border-like' cells [22]. The distribution of YFP-TIP1;2 is therefore radically different to that observed for its paralogue, TIP1;1-YFP, which has the widest pattern of expression but is excluded from the root tip, including the root cap (Fig. 1, panels A to M).

At the subcellular level, YFP-TIP1;2 localises to the endoplasmic reticulum (ER) of young root cap cells (Fig 3e: note the characteristic reticular pattern and the nuclear envelope; see also Additional file 1B). The chimeric protein is mostly found on the tonoplast of elongated lateral root cap cells (Fig. 3D). This is likely to reflect different stages of TIP1;2 trafficking in cells of different ages, rather than impaired capacity to reach the tonoplast. This is further demonstrated by the fact that in the epidermis of cotyledonary cells, where TIP1;2 is uniformly expressed, the fusion protein appears to localise to the tonoplast (Additional file 1C-D).

### TIP2;1 is localised in lateral root primordia

**Figure 4:**
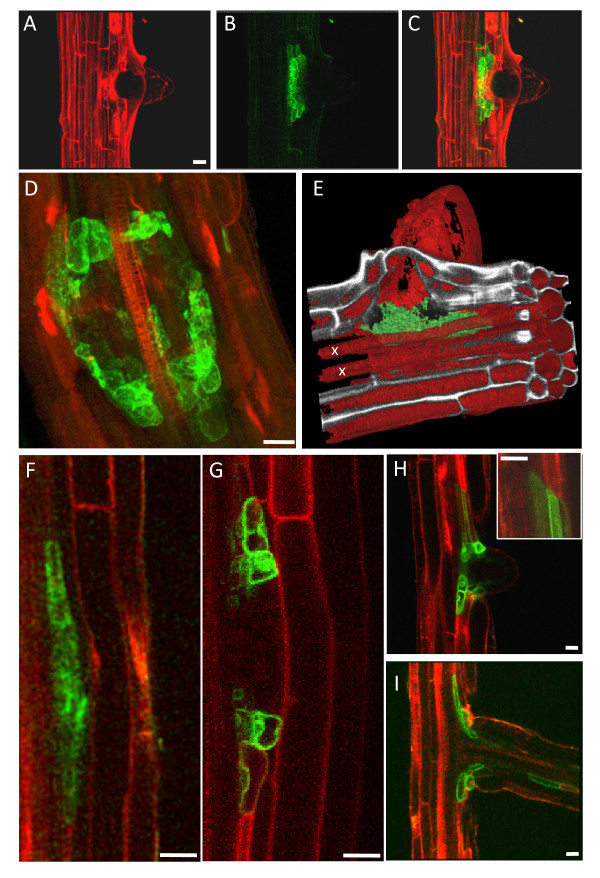
**TIP 2;1-YFP expression in lateral root primordia**. A-E: 8-day old roots from TIP2;1-YFP transgenic seedlings were excised, stained with propidium iodide and visualised by CLSM. Stacks of 80 optical z sections (1 μm step-size) were collected from mature root axes. The images show representative results for this construct. Maximal projections of the z-stacks are shown, with the individual signals for YFP (A), propidium iodide (B) or the merged signals (C and D). E: The fluorescent traces from YFP (green) and propidium iodide (red) from the image stack in panels (A-C) was reconstructed, segmented and rendered in 3D with Mimics 12.1. Note that the TIP2;1-YFP-expressing cells are in close proximity to the xylem (labelled with x). F-I: Roots from 8-day old transgenic seedlings expressing TIP2;1-YFP (green) and TIP2;3-RFP (red) were imaged. Sequential stages of lateral root development are shown. Inset in H: note the boundary between pericycle cells expressing TIP2;3-RFP (top) and TIP2;1-YFP (bottom). Scale bars: 20 μm.

We have previously shown that TIP2;1 expression becomes detectable in old root regions nearing the hypocotyl, and is then widespread in hypocotyl and cotyledonary leaves [14]. We did not initially notice expression in young roots, but closer analysis revealed that in 8-day old roots TIP2;1-YFP has a very specialised expression pattern (Fig. 4). The YFP signal is detected in a ring-like cluster at the base of emerging lateral roots (Fig. 4, panels A-D). In very early lateral root primordia (LRP), TIP2;1 expression is detectable in 2-4 cells at the LRP. As the LRP grows further, the number of cells expressing TIP2;1-YFP increases but remains confined to a cluster underlying the base the lateral root (Fig. 4, panels F-I). In rare cases, when the lateral root is fully emerged, the expression of TIP2;1-YFP can extend to some cells within the lateral root axis (Fig 4I). Co-labelling with propidium iodide shows that the TIP2;1 expressing cells are located in close proximity to the xylem (fig. 4E), suggesting a pericycle localisation. Co-expression with TIP2;3-RFP, which we found to be enriched in the pericycle (Fig. 2, panels D, E, H) confirms that TIP2;1-YFP expression originates from pericycle cells (Fig. 4, panels F-I). This indicates that the initial expression of TIP2;1-YFP is likely to occur in the LRP founder cells. Remarkably, the expression of TIP2;1-YFP and TIP2;3-RFP appears to be mutually exclusive, with a clear boundary between cells expressing one or the other isoform (Fig. 4H, inset; see Additional file 2 for individual channels).

### Overlapping TIP isoforms are mostly detectable at the central vacuole tonoplast

**Figure 5:**
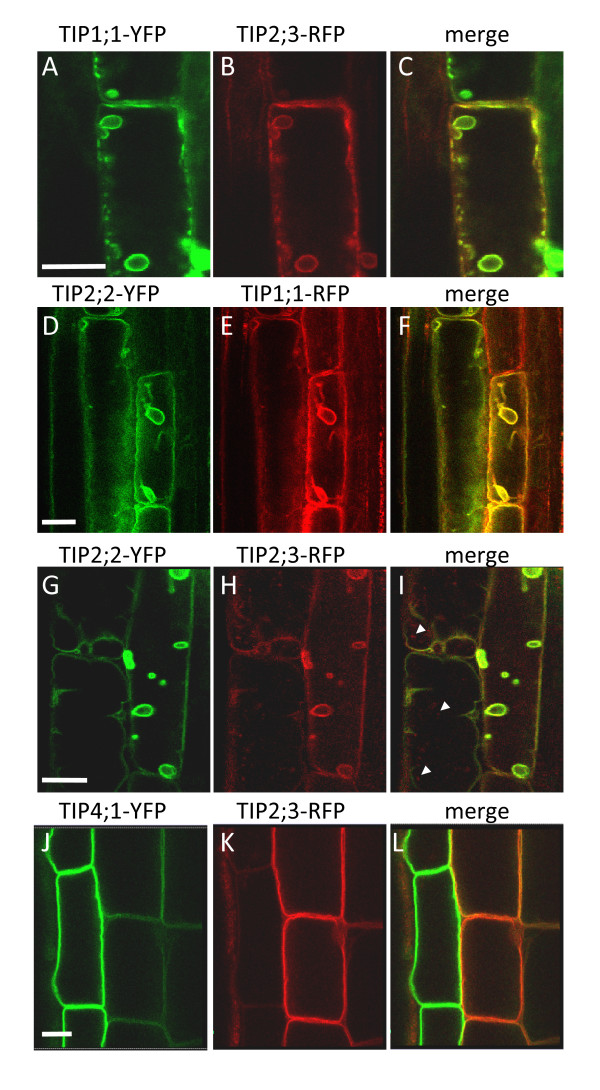
**Overlapping TIP isoforms are mainly detected at the tonoplast of the central vacuole**. Transgenic seedlings co-expressing the indicated TIP-YFP and TIP-RFP constructs were grown for 8 days on MS medium--agar plates. Roots were excised and visualised by CLSM. (A, D, G, J): YFP fluorescence (green); (B, E, H, K): RFP fluorescence (red); (C, F, I, L): merged images. Arrowheads in panel I indicate structures labelled by TIP2;3-RFP but not TIP2;2-YFP. Scale bars:10 μm.

We have shown that the various TIP isoforms under study present diverse tissue specificity within roots. Several isoforms, however, are co-expressed in certain tissues, namely TIP1;1, TIP2;2, TIP2;3, and TIP4;1. In order to ascertain whether these isoforms were specific to distinct vacuolar compartments, we focused on the subcellular localisation of selected pairs of the above isoforms, tagged with different spectral variants of fluorescent proteins and co-expressed in transgenic Arabidopsis (Fig. 5).

The individual TIP expression patterns in double transgenic lines mirrored those observed in the lines expressing individual isoforms (Additional file 3). The widespread TIP1;1-YFP and TIP2;3-RFP are co-expressed in epidermis, cortex and pericycle cells (Fig. 2). In these tissues, both proteins are detected on the tonoplast of the central vacuole (Fig. 5A-C). Both the tonoplast and the smaller, bulb-like vacuolar structures [18] are labelled. Likewise, TIP2;2-YFP and TIP1;1-RFP mostly label the same tonoplast in the cell layers where they are co-expressed (Fig. 5D-F). TIP2;2-YFP and TIP2;3-RFP, which almost overlap in root tissues (Fig. 2), are also co-localised on tonoplast and 'bulbs' (Fig. 5G-I). Occasionally, TIP2;3-RFP highlighted smaller vesicular structures that did not appear to contain TIP2;2-YFP (Fig 5I, arrowheads). The nature of these structures was not investigated further. Finally, TIP4;1-YFP, which is restricted to epidermis and cortex (Fig. 2F), co-localises with TIP2;3 in those tissues (Fig. 5J-L). Note that the relative abundance of these two isoforms mirrors the pattern observed in single isoform localisation, with TIP4;1 expression being strongest in the epidermis and weaker in the cortex (Fig. 2F), and TIP2;3 expression being stronger in cortex but weaker in epidermis (Fig. 2, panels D-E).

Taken together, these co-expression results indicate that each TIP isoform-fluorescent protein fusion we analysed is predominantly found at the central vacuole tonoplast in Arabidopsis root tissues.

## Discussion

We have produced a complete expression map for all members of the TIP family that are present in Arabidopsis root tissues, including isoforms not previously studied. The use of XFP fusions to TIP genomic sequences allowed us to investigate both the tissue specificity and the subcellular localisation of these proteins.

In general, our fluorescent reporter - TIP localisation data correlate well with the relative TIP transcript levels, as observed by microarray analysis [15,23,24], with the exception of TIP1;2. TIP1;2 is indeed the isoform with the highest level of mRNA expression in the root cap [23], which matches our observations (Fig. 3). However, transcript levels for TIP1;2 have also been shown to be almost as high as TIP1;1 throughout the root axis [15,23,24]. We can only speculate at this stage that post-transcriptional control processes prevent TIP1;2-YFP protein from being detectable in these tissues.

TIP1;1 is the most widely expressed isoform along the root axis. TIP2;2 and 2;3 have very similar expression patterns, with their expression being low in the endodermis, but high in the xylem pole pericycle. It appears that TIP2;1 becomes strongly expressed in the pericycle when this undergoes differentiation to form the lateral root primordium (Fig. 4). This narrow range of localisation of TIP2;1 is intriguing. Transcripts of the maize aquaporin ZmTIP1 were also localised in the lateral root by in situ hybridisation, but every cell in the LRP seemed to contain the transcript [25]. TIP2;1 can therefore be considered an additional marker for the Arabidopsis LRP margins, alongside the auxin efflux carriers Pin4 and Pin6 [26] and the transcription factor CUC3 [27], which have a similar localisation. It will be interesting to study the specific role of TIP2;1 in these cells and determine why this stage of lateral root development demands such a precise TIP isoform activation.

As a general observation, we could not detect expression of any of the TIP-XFP fusions under study in the root tip meristem. This lack of expression was previously reported for TIP1;1 both by histochemical detection of GUS fusions [5] and YFP tagging [14,19]. It is of course possible that expression levels of our fusions are too low in this region to be detected by confocal microscopy. However, the fact that the more sensitive histochemical GUS staining also fails to detect expression of TIP1;1, which microarray data indicate is the most abundantly transcribed isoform in roots [16], strongly suggest that the protein is not expressed in the root meristem. This is in contrast with data from other species such as pea and barley, where TIPs have been located in isolated root tip cells [10,12] and in root tip sections by immunohistochemical methods [28]. Analysis in the Olbrich et al. study was performed on 3-day old seedlings. At the same age in Arabidopsis seedlings we could already detect all the TIP isoforms described in this study, with the exception of TIP2;1. However their expression pattern was already the same as observed at 8 days (data not shown). We therefore resolved to present results at 8 days, when the complete set of root TIPs is detectable.

This lack of observable expression in root tips makes it difficult to perform meaningful comparisons between the vacuolar complement of Arabidopsis root tip cells and that of other plant species.

TIP-YFP expression was also not detected in the root vasculature, regardless of the developmental stage. This mirrors observations in barley and pea root sections, where the stele was not labelled by TIP antisera [28]. While it is easy to rationalise the absence of a vacuole in the xylem cells, which underwent autolysis, and in mature sieve elements, which lack true vacuoles (reviewed in [29]) it was somewhat surprising not to find TIPs in the companion and parenchima cells. We think it unlikely that this lack of detection is caused by a loss of sensitivity by the confocal microscope detectors in the inner layers of the roots, because both propidium iodide staining and YFP signal are easily detected in the xylem and xylem pole pericycle, respectively (Fig. 2 and Additional file 3). In addition, we could easily detect 35S::TIP2;1-YFP in the vascular tissue using the same settings (Additional file 4). Accordingly, Boursiac et al [24] recently showed that constitutively expressed TIP1;1-GFP and TIP1;2-GFP clearly label the vascular tissue [24].

Recently it has been shown that Arabidopsis knockout mutants lacking TIP1;1, TIP1;2, or both isoforms, do not have any major defects [7,19]. This is in contrast with drastic defects observed in Arabidopsis upon downregulation of TIP1;1 by RNAi [30]. A possible explanation for the latter result is off-target silencing in the RNAi lines [7]. Our data provide a rationale for the lack of a macroscopic phenotype in the double TIP1 knockouts observed by Schussler et al. We have shown that, in roots, expression of TIP1;1 and TIP1;2 does not appear to overlap, with TIP1;1 being expressed in epidermis, cortex, endodermis and perycycle starting from the elongation zone, and TIP1;2 being restricted to the root cap. As TIP1;1 and 1;2 show different tissue specificities, it seems unlikely that they are reciprocally redundant. In addition, we have shown that other TIP isoforms, namely TIP2;2, TIP2;3 and TIP4;1 would still be present in the tissues lacking TIP1;1 (Fig. 2). It is therefore possible that these remaining isoforms compensate for the lack of TIP1;1 in the knockout. On the other hand, the effect of the absence of TIP1;2 from the root cap may be subtle and may have gone undetected under the experimental conditions adopted for the whole-plant analysis of the double mutants. A lack of phenotype in the aerial parts of the single knockout plants may be explained by the fact that both TIP1;1 and TIP1;2 are expressed in leaves [14] and Additional file 2) and may well be acting redundantly there. As for the double knockout, redundancy may be afforded by TIP2;1 [14] and TIP2;2 [16], which are also expressed in leaves.

## Conclusion

We have identified novel patterns of expression of TIP isoforms in Arabidopsis roots. This information may provide a useful starting point for a more targeted approach to dissect the function of individual TIP isoforms in root tissues. It also provides the foundation for further analysis of the intracellular targeting of different TIPs.

## Methods

### Recombinant DNA and generation of transgenic plants

The constructs encoding native TIP1;1-YFP and native TIP2;1YFP have been described previously [14].

A full list of primers designed to amplify the genomic sequences of the root-expressed TIPs is shown in Additional file 5. Each TIP genomic sequence, including either the complete promoter region (up to the UTR of the gene immediately upstream in the chromosome) or 1.5 Kb of the promoter (if longer than 1.5 Kb), plus 5' UTR and introns, was amplified from total genomic DNA from *Arabidopsis thaliana *Columbia ecotype. Primers included restriction sites KpnI at the 5' and XhoI at the 3' of the target sequences. Amplified fragments were cloned into the KpnI and XhoI sites of pGREEN0029, upstream of a XhoI/SacI fragment containing the YFP coding sequence and the OCS 3' terminator fragment. A similar strategy was adopted to fuse TIP sequences to RFP, but in this case the forward primers included both KpnI and SacI restriction sites, generating a TIP-RFP cassette that could be mobilized with SacI. To obtain pairwise TIP-YFP/TIP-RFP combinations, selected TIP-RFP cassettes were excised with SacI and ligated into TIP-YFP vectors linearised with SacI, giving rise to constructs harbouring both reporter genes in a tandem. All the chimeric constructs were introduced into strain C58 of Agrobacterium tumefaciens harbouring the pSoup vector [31]. Arabidopsis plants were then transformed using the floral dip method [32].

### Confocal analysis and image processing

About 30 seeds from at least 4 independent transgenic lines per construct were germinated onto agar plates containing half-strength Murashige and Skoog (MS) Basal Medium (Sigma-Aldrich) and grown for 8 days at 22°C, in a 16:8 light:dark regime. Roots were excised, mounted in half-strength liquid MS medium and immediately observed with a Leica TSC SP5 confocal laser scanning microscope, using either a 10× (NA 0.3) air or a 63× (NA 1.4) oil immersion objective. In some cases roots were preincubated for 2 min in 10 μg/ml propidium iodide, diluted in half-strength MS medium. YFP was excited at 514 nm and detected in the 525 to 550 nm range. RFP was excited at 561 nm and detected in the 553 to 638 nm range. Propidium iodide was excited at 561 nm and detected in the 650 to 720 nm range. Simultaneous detection of YFP and RFP or YFP and propidium iodide was performed by combining the settings indicated above in the sequential scanning facility of the microscope, as instructed by the manufacturer.

3D reconstruction of z-stacks of optical sections was performed with the Leica LAS-AF Lite free software (Leica Microsystems, Germany). Segmentation analysis and 3D rendering were performed with Mimics 12.1 (Materialise N.V., Leuwen, Belgium).

## Abbreviations

CLSM: confocal laser scanning microscopy; ER: endoplasmic reticulum; PI: propidium iodide; TIP: tonoplast intrinsic protein.

## Authors' contributions

SG generated the majority of the constructs and transgenic plants and performed the bulk of the confocal analysis. MS produced the TIP1;2-RFP and 35S:TIP2;1 constructs and transgenic lines and performed confocal analysis. PH produced the native TIP1;1-YFP and TIP2;1 YFP constructs and transgenic plants and performed confocal analysis. RK performed the 3D image analysis in MIMICS. LF designed the experimental programme, gave technical and intellectual guidance and wrote the manuscript. All authors read and approved the final manuscript.

## Supplementary Material

Additional file 1 **Expression and subcellular localisation of TIP1;2**. 8-day old seedlings expressing YFP-TIP1;2 were visualised by CLSM. A: 10× magnification of a lateral root. The signals from YFP fluorescence (green) and propidium iodide fluorescence (red) are merged. B: single root cap cell with TIP1;2-YFP showing typical ER labelling. C-D: epidermal cells in cotyledons where TIP1;2-YFP shows typical tonoplast labelling (green). Red: chlorophyll autofluorescence (excitation 514 nm, detection 600-650 nm). Scale bars: A, 100 μm; B and D, 5 μm; C, 20 μm. Click here for file

Additional file 2 **Mutually exclusive expression of TIP2;1 and TIP2;3 in lateral root primordia**. Roots from 8-day old transgenic seedlings expressing TIP2;1-YFP (green) and TIP2;3-RFP (red) were visualised by CLSM. Scale bar, 20 μm. Click here for file

Additional file 3 **Co-expression of selected TIP-XFP pairs**. Transgenic seedlings co-expressing the indicated TIP-YFP and TIP-RFP constructs were grown for 8 days on MS medium-agar plates. Roots were excised and visualised by CLSM. Stacks of 80 optical z sections (1 μm step-size) were collected from root axes at the differentiation zone. The images show representative results for each construct. Each panel shows the xz projection of the whole image stack, revealing the cross section of the root axis. Click here for file

Additional file 4 **Constitutively expressed TIP2;1-YFP is detectable in every root tissue**. Roots from 8-day old transgenic seedlings expressing 35S::TIP2;1-YFP (green) were excised, stained with propidium iodide (red) for 2 min and visualised by CLSM. A: stacks of 80 optical z sections (1 μm step-size) were collected from root axes at the differentiation zone. The images show representative results for this construct. The signals from YFP fluorescence (green) and propidium iodide fluorescence (red) are merged. B-D: single optical section through the vascular tissue, indicating that constitutive expression of TIP2;1 is easily detectable in these cell types. B: YFP, C, propidium iodide, D, merged images. Scale bar, 10 μm. Click here for file

Additional file 5 **Primers used in this study**. The diagram indicates the target sequences for the indicated primers in the final constructs. Restriction sites are shown in bold. Click here for file
