# Supplementary figures and images for: In vivo imaging of the tonoplast intrinsic protein family in Arabidopsis roots
Source: BMC Plant Biol. 2009 Nov 18;9:133. doi: 10.1186/1471-2229-9-133 (PMC2784467; doi:10.1186/1471-2229-9-133)

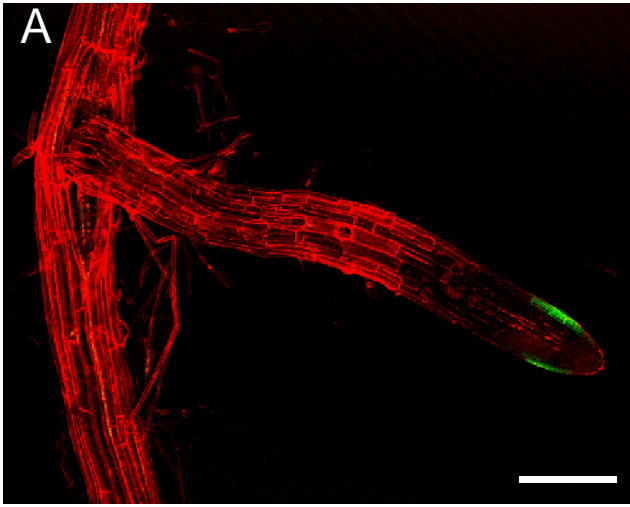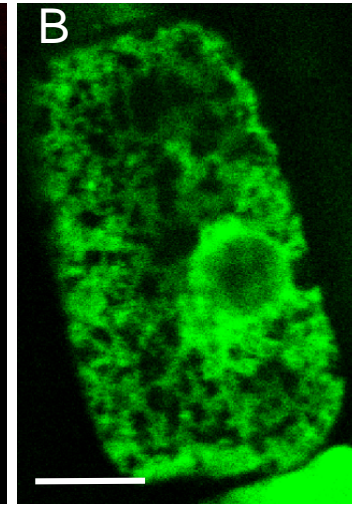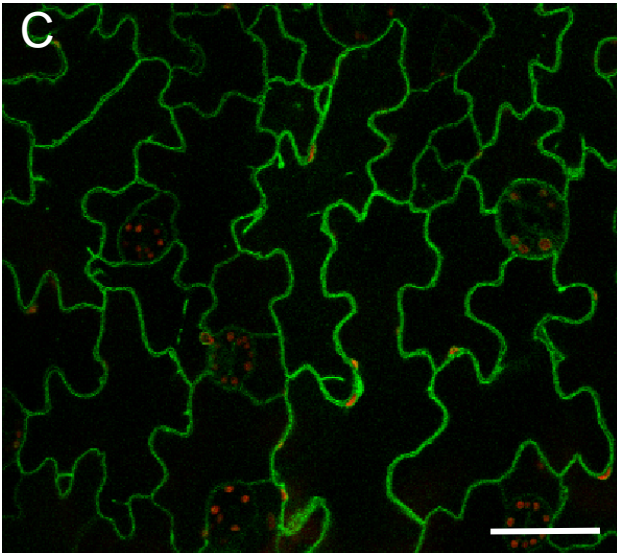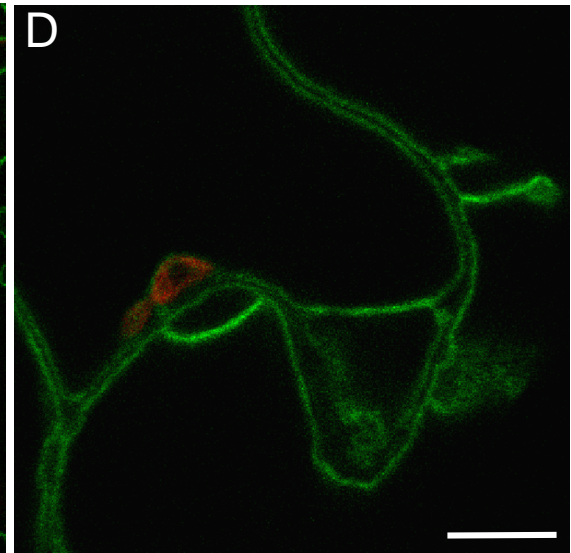

Supplement: Additional file 1 — Expression and subcellular localisation of TIP1;2. 8-day old seedlings expressing YFP-TIP1;2 were visualised by CLSM. A: 10× magnification of a lateral root. The signals from YFP fluorescence (green) and propidium iodide fluorescence (red) are merged. B: single root cap cell with TIP1;2-YFP showing typical ER labelling. C-D: epidermal cells in cotyledons where TIP1;2-YFP shows typical tonoplast labelling (green). Red: chlorophyll autofluorescence (excitation 514 nm, detection 600-650 nm). Scale bars: A, 100 μm; B and D, 5 μm; C, 20 μm. [file 1471-2229-9-133-S1.PDF]

TIP2;3-RFP

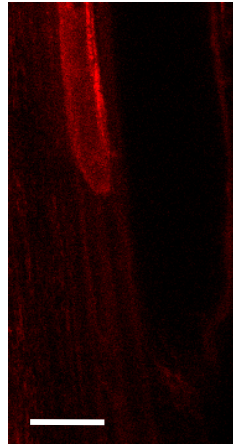

TIP2;1-YFP

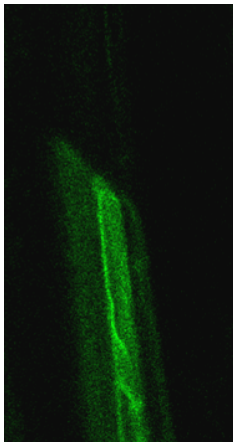

merge

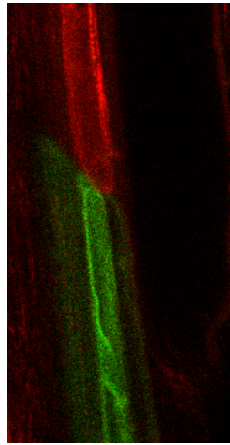

Supplement: Additional file 2 — Mutually exclusive expression of TIP2;1 and TIP2;3 in lateral root primordia. Roots from 8-day old transgenic seedlings expressing TIP2;1-YFP (green) and TIP2;3-RFP (red) were visualised by CLSM. Scale bar, 20 μm. [file 1471-2229-9-133-S2.PDF]

TIP2;2-YFP

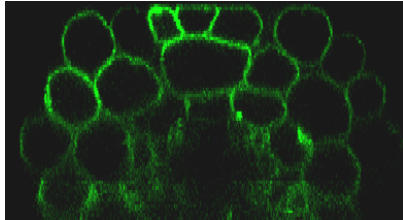

TIP2;3-RFP

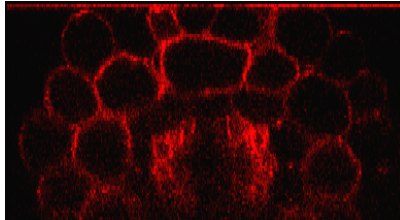

merge

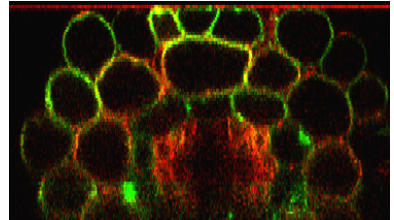

TIP2;2-YFP

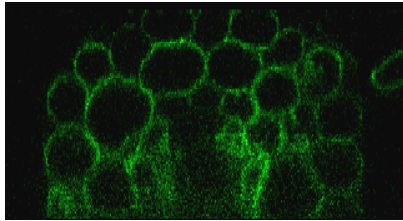

TIP1;1-RFP

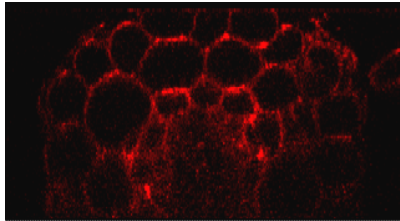

merge

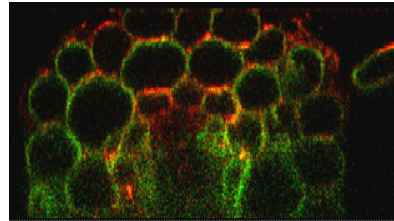

TIP1;1-YFP

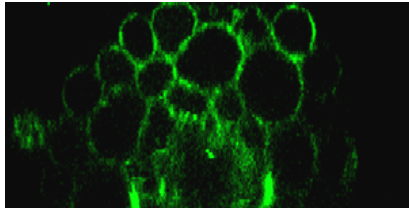

TIP2;3-RFP

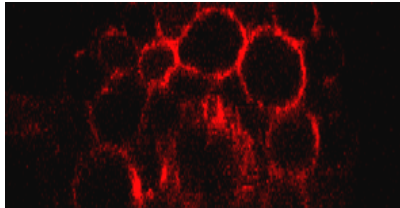

merge

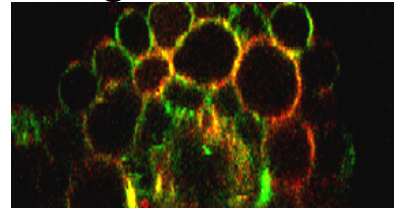

TIP4;1-YFP

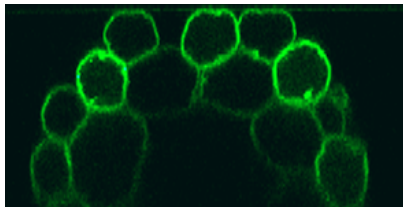

TIP2;3-RFP

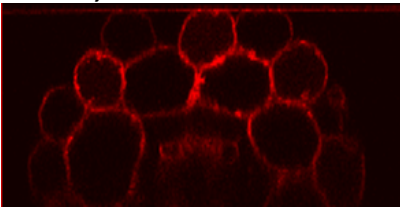

merge

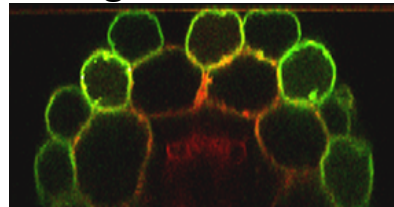

Supplement: Additional file 3 — Co-expression of selected TIP-XFP pairs. Transgenic seedlings co-expressing the indicated TIP-YFP and TIP-RFP constructs were grown for 8 days on MS medium-agar plates. Roots were excised and visualised by CLSM. Stacks of 80 optical z sections (1 μm step-size) were collected from root axes at the differentiation zone. The images show representative results for each construct. Each panel shows the xz projection of the whole image stack, revealing the cross section of the root axis. [file 1471-2229-9-133-S3.PDF]

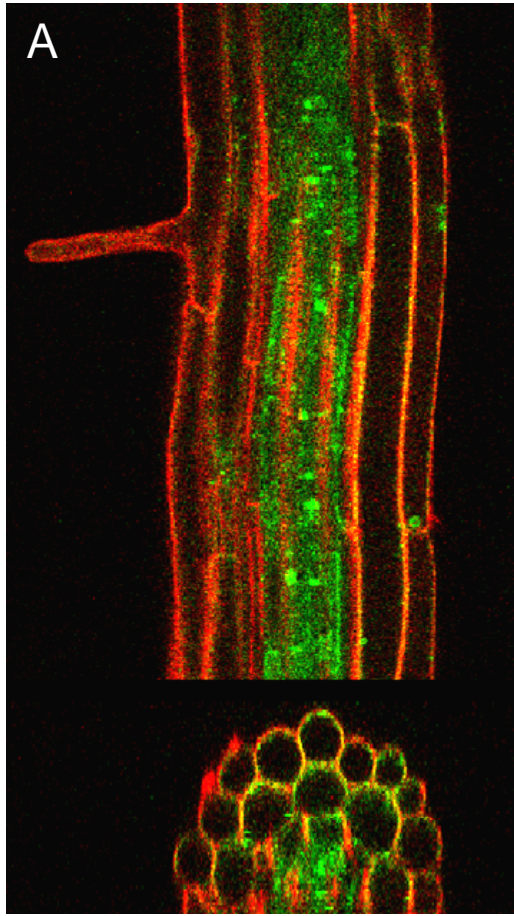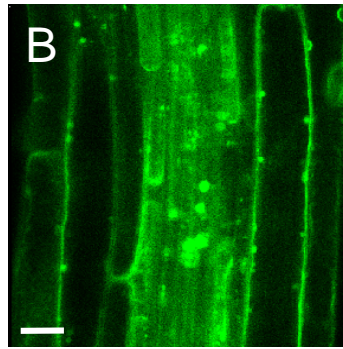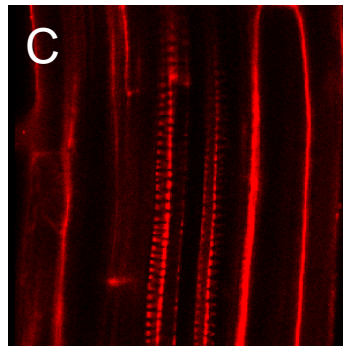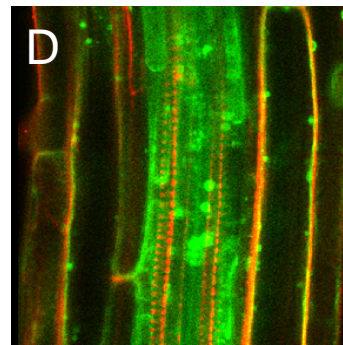

Supplement: Additional file 4 — Constitutively expressed TIP2;1-YFP is detectable in every root tissue. Roots from 8-day old transgenic seedlings expressing 35S::TIP2;1-YFP (green) were excised, stained with propidium iodide (red) for 2 min and visualised by CLSM. A: stacks of 80 optical z sections (1 μm step-size) were collected from root axes at the differentiation zone. The images show representative results for this construct. The signals from YFP fluorescence (green) and propidium iodide fluorescence (red) are merged. B-D: single optical section through the vascular tissue, indicating that constitutive expression of TIP2;1 is easily detectable in these cell types. B: YFP, C, propidium iodide, D, merged images. Scale bar, 10 μm. [file 1471-2229-9-133-S4.PDF]
