# Supplementary material for: In vivo imaging of the tonoplast intrinsic protein family in Arabidopsis roots
Source: BMC Plant Biol. 2009 Nov 18;9:133. doi: 10.1186/1471-2229-9-133 (PMC2784467; doi:10.1186/1471-2229-9-133)
Supplement: Additional file 5 — Primers used in this study. The diagram indicates the target sequences for the indicated primers in the final constructs. Restriction sites are shown in bold. [file 1471-2229-9-133-S5.PDF]

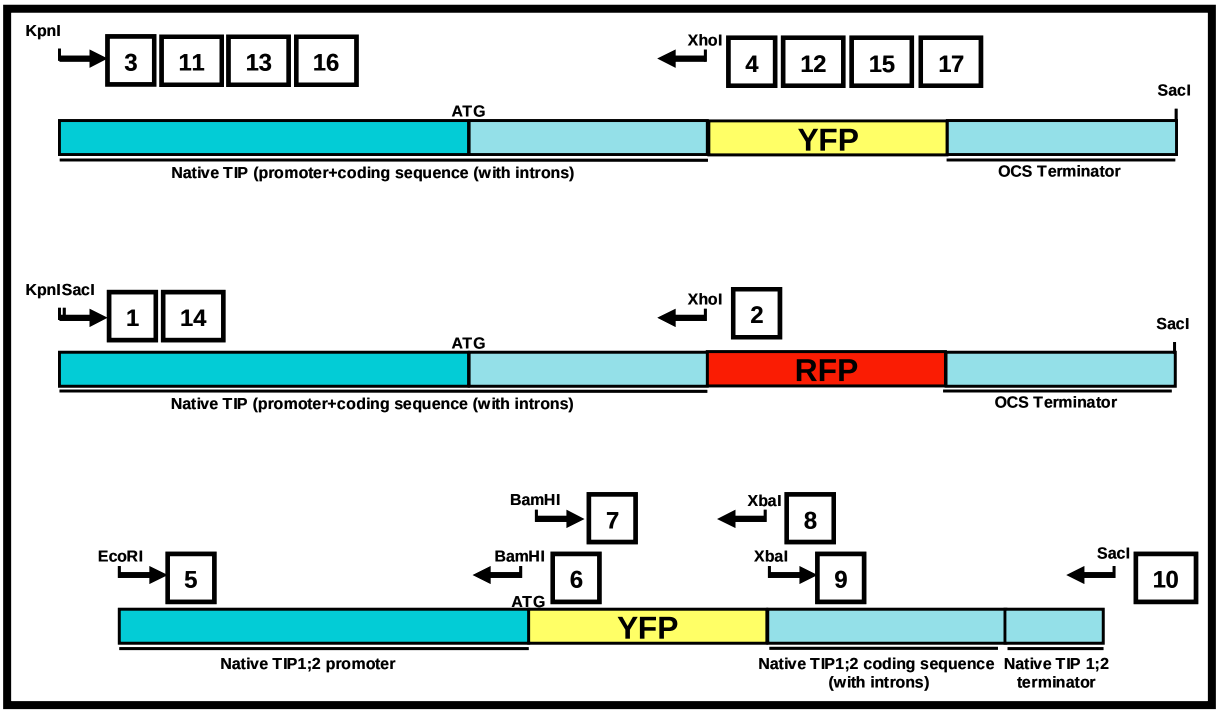

1	TIP1-1kpnIsacI_F	CAGATA**GGTACCGAGCTC**CAATGGATCATCCACCTAAC
2	TIP1-1xhoI_R	CACATA**CTCGAG**GTAGTCTGTGGTTGGGAGC
3	TIP1-2kpnI_F	CACATA**GGTACC**TACACATTAATAGAG
4	TIP1-2xhoI_R	CACATA**CTCGAG**GTAATCGGTGGTAGGCAATTG
5	TIP1-2(P)ecoRI_F	CACATA**GAATTC**CGGTACCTACACATTAATAGAG
6	TIP1-2(P)bamHI_R	CACATA**GGATCC**GATCGGAGAAAGATTTAACG
7	YFPbamHI_F	CACATA**GGATCC**ATGGGCAGCAAGGGCGAGGAGC
8	YFPxbaI_R	CACATA**TCTAGA**GATCACCTTGTACAGCTCGTCC
9	TIP1-2(C)xbaI_F	CACATA**TCTAGA**CCGACCAGAAACATCGCCATTGG
10	TIP1-2(T)sacI_R	CACATA**GAGCTC**ACAAAATGAAATCCGAATGACG
11	TIP2-2kpnI_F	TT**GGTACC**TGTTTGACGTGACTGTGGTA
12	TIP2-2xhoI_R	TT**CTCGAG**AGGGTAGCTTTCTGTGGTGGGAGC
13	TIP2-3kpnI_F	CACATA**GGTACC**GGGTTTAGCAATCTTTCAG
14	TIP2-3kpnIsacI_F	CACATA**GGTACCGAGCTC**GGGTTTAGCAATCTTTCAG
15	TIP2-3xhoI_R	CACATA**CTCGAG**CACTCGGATCTCACGGGTTTCTAC
16	TIP4-1kpnI_F	CACATA**GGTACC**AACTCGTCCATTCTGCATGTC
17	TIP4-1xhoI_R	CACATA**CTCGAG**ATTCAACAATGGTTGCTCGTCG
